# Comparison of chemical compositions, antioxidant activities, and acetylcholinesterase inhibitory activities between coffee flowers and leaves as potential novel foods

**DOI:** 10.1002/fsn3.3126

**Published:** 2022-11-05

**Authors:** Xiaojing Shen, Fanqiu Nie, Haixian Fang, Kunyi Liu, Zelin Li, Xingyu Li, Yumeng Chen, Rui Chen, Tingting Zheng, Jiangping Fan

**Affiliations:** ^1^ College of Food Science and Technology, College of Science Yunnan Agricultural University Kunming China; ^2^ Yunnan Key Laboratory of Pharmacology for Natural Products Kunming Medical University Kunming China; ^3^ Yunnan Organic Tea Industry Intelligent Engineering Research Center Key Laboratory of Intelligent Organic Tea Garden Construction in Universities of Yunnan Province Kunming China; ^4^ Quality Standardizing and Testing Technology Institute, Yunnan Academy of Agricultural Sciences Kunming China; ^5^ College of Wuliangye Technology and Food Engineering Yibin Vocational and Technical College Yibin China; ^6^ Research Platform for Innovation and Utilization of Medicine Food Homology and Fermented Food Yibin Vocational and Technical College Yibin China; ^7^ Pu'er Institute of Pu‐erh Tea Pu'er China

**Keywords:** acetylcholinesterase inhibitory activity, antioxidant activity, chemical composition, coffee flower, coffee leaf

## Abstract

This study aimed to compare chemical compositions, antioxidant activities, and acetylcholinesterase inhibitory activities of coffee flowers (ACF) and coffee leaves (ACL) with green coffee beans (ACGB) of *Coffea Arabica* L. The chemical compositions were determined by employing high‐performance liquid chromatography–mass spectroscopy (HPLC–MS) and gas chromatography–mass spectroscopy (GC–MS) techniques. Antioxidant effects of the components were evaluated using DPPH and ABTS radical scavenging assays, and the ferric reducing antioxidant power (FRAP) assay. Their acetylcholinesterase inhibitory activities were also evaluated. The coffee sample extracts contained a total of 214 components identified by HPLC‐MS and belonged to 12 classes (such as nucleotides and amino acids and their derivatives, tannins, flavonoids, alkaloids, benzene, phenylpropanoids, and lipids.), where phenylpropanoids were the dominant component (>30%). The contents of flavonoids, alkaloids, saccharides, and carboxylic acid and its derivatives in ACF and ACL varied significantly (*p* < .05) compared to similar components in ACGB. Meanwhile, 30 differentially changed chemical compositions (variable importance in projection [VIP] > 1, *p* < .01 and fold change [FC] > 4, or <0.25), that determine the difference in characteristics, were confirmed in the three coffee samples. Furthermore, among 25 volatile chemical components identified by GC–MS, caffeine, n‐hexadecanoic acid, 2,2′‐methylenebis[6‐(1,1‐dimethylethyl)‐4‐methyl‐phenol], and quinic acid were common in these samples with caffeine being the highest in percentage. In addition, ACL showed the significantly highest (*p* < .05) DPPH radical scavenging capacity with IC_50_ value of 0.491 ± 0.148 mg/ml, and acetylcholinesterase inhibitory activity with inhibition ratio 25.18 ± 2.96%, whereas ACF showed the significantly highest (*p* < .05) ABTS radical scavenging activity with 36.413 ± 1.523 mmol trolox/g Ex. The results suggested that ACL and ACF had potential values as novel foods in the future.

## Graphical abstract

The chemical compositions of coffee flowers and leaves Arabic Coffea from Yunnan province, China were compared with green coffee beans by HPLC–MS and GC–MS. Antioxidant and acetylcholinesterase inhibitory activities were higher than green coffee beans in vitro. Thirty differentially changed chemical compositions were confirmed.

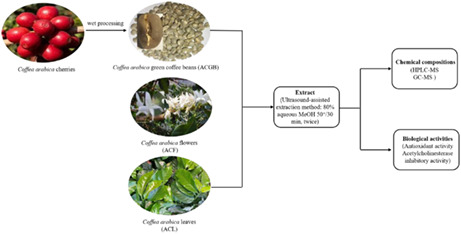

## 1 INTRODUCTION

Coffea is a genus of the family Rubiaceae, used to make coffee beverages, which are on the top among the three beverages in the world, due to their rich and complex flavor and medicinal values. As an important plantation crop, Coffea is grown in more than 80 countries around the world (Godos et al., 2014). Coffea cultivating areas cover about 10.6 million ha of land and are mainly distributed in the tropics, such as Brazil, Colombia, Venezuela, Paraguay, Indonesia, India, Ethiopia, and Mexico (Silva et al., 2013). A large number of coffee by‐products including grounds, silver skins, husks, flowers, and leaves of coffee are produced by the global coffee plantation and processing industries. Some coffee by‐products are already used as food in Europe and non‐EU‐member countries, or have been applied for authorization as novel food already (Klingel et al., 2020).

Coffee by‐products are beneficial for human health, owing to the presence of some natural bioactive compounds, such as phenolic acids, flavonoids, terpenes, and alkaloids. These bioactive compounds show antioxidant and hepato‐ and neuro‐protective activities (Chen et al., 2018; Martinez‐Saez & Dolores del Castillo, 2019; Rebollo‐Hernanza et al., 2019). Coffee by‐products, which can be important sources of natural functional compounds in the future, will contribute to the development of functional compounds and circular economy (Comunian et al., 2021; Panwar et al., 2021). In addition, coffee by‐products can be recycled to produce value‐added products in bioenergy segment. However, the increasing production of solid residues of coffee by‐products originating from annual coffee production has brought about environmental concerns.

Coffee flowers, as the primary coffee by‐products are usually abandoned in coffee cultivation, have received growing public attention and research interests for their potential human health benefits due to their various phytochemicals (Nguyen et al., 2019; Pinheiro et al., 2021). Pinheiro et al. (2021) suggested that the flowers of *C. Arabica* and *C. Conilon* possessed antioxidant properties. Thus, coffee flowers can be potentially used for research and development of bioactive compounds focusing on human health. Coffee leaves were widely used as medicine and beverages in some countries and regions consuming tea as their primary beverage (Chen et al., 2018). The health benefits of bioactive components in coffee leaves have been reported by several researchers (Campa et al., 2012; Chen et al., 2018). The chemical composition of coffee leaves consists of alkaloids, flavonoids, phenolic acids, terpenes, and so on, responsible for antioxidation, anti‐inflammatory, antitumor, antidiabetic, and neuroprotective activities (Chen et al., 2018).

Studies had found that oxidative stress is one of the most important mechanisms of cellular senescence and increased frailty, resulting in several age‐linked, noncommunicable diseases (Martemucci et al., 2022). Antioxidants can protect cells against free radical damage, as well as help in reducing the risk of many chronic diseases, such as Alzheimer's disease (AD) (Singh et al., 2020). In addition, AD is related to the decrease of the neurotransmitter acetylcholine (ACh) levels (Zavala‐Ocampo et al., 2022). Based on the cholinergic hypothesis cholinesterase inhibitors are used to re‐establish the levels of acetylcholine in the brain (Sahibzada et al., 2022). Therefore, antioxidant and acetylcholinesterase inhibitory activities are the basis for further studies in the development of therapies for neurodegenerative disorders. Eicosanoyl‐5‐hydroxytryptamide, caffeic acid, and caffeine both showed a beneficial therapeutic effect in a rat model of sporadic AD. (Asam et al., 2017; Rezg et al., 2008; Zeitlin et al., 2011).

**FIGURE 1:**
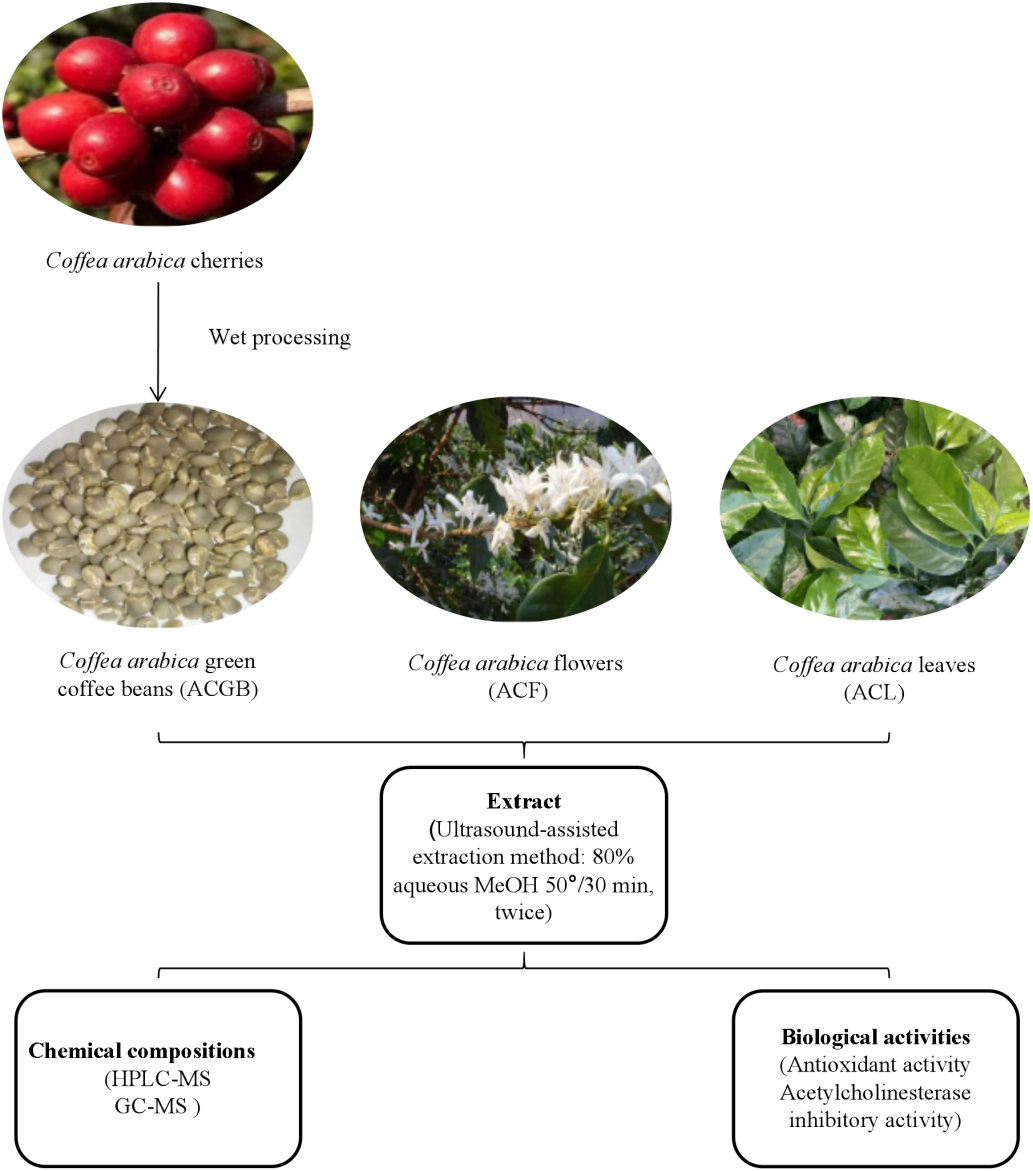
Experimental design

Yunnan province, the main coffee plantation area in China, undertakes 99% of Chinese coffee plantations. A large number of coffee flowers and leaves were discarded annually. The aim of this study is to provide data for further development and utilization of coffee by‐products as well as to enhance the value of coffee. The experimental design of the study is shown in Figure 1. The identification of chemical compounds in coffee flowers (ACF), leaves (ACL), and green coffee beans (ACGB) from *C. Arabica* was done by high‐performance liquid chromatography–mass spectroscopy (HPLC‐MS) and gas chromatography–mass spectroscopy (GC–MS). Furthermore, multivariate statistical techniques were used to investigate the differentially varying chemical compositions of the coffee samples. Their antioxidant activities were compared by the 2,2‐diphenyl‐1‐picrylhydrazyl (DPPH), 2,2′‐azino‐bis (3‐ethyl‐benzothiazoline‐6‐sulphonic acid) (ABTS) radical scavenging, and ferric reducing antioxidant power (FRAP) assays. Their acetylcholinesterase inhibitory activities were evaluated as well.

## 2 MATERIALS AND METHODS

### 2.1 Plant material and reagents

Coffee flowers, coffee leaves, and fresh coffee cherries from *C. Arabica* were harvested and collected from Baoshan City, Yunnan Province, China. Coffee flowers and leaves were crushed and dried at room temperature. Green coffee beans were obtained by wet processing. The voucher specimens (No. ACBS1‐3) were obtained from College of Science, Yunnan Agricultural University.

Rutin, DPPH, ABTS, and tripyridyltriazine (TPTZ) were purchased from Shanghai Ryon Biological Technology Co., Ltd. Trolox was obtained from HeFei BoMei Biotechnology Co., Ltd. All reagents were of chemical purity.

High‐performance liquid chromatography grade methanol and acetonitrile were sourced from Merck KgaA, and formic acid was purchased from Xiya Reagent.

### 2.2 Sample preparation

The dried samples were extracted using an ultrasound‐assisted extraction method as follows: 25.0 g of the powdered samples were extracted with 200 ml of 80% MeOH aqueous (*V*/*V*) for 30 min. After extraction, the resulting solution was filtered with a filter paper. The residue obtained was washed with 100 ml of MeOH subsequently and was extracted again. The filtrates were then combined and concentrated using a YaRong rotary evaporator (Shanghai, China) at 50°C and finally freeze‐dried. Afterward, the extracts were re‐dissolved and stored at 4°C.

### 2.3 HPLC–MS analysis

Sample extracts were analyzed with Q‐Exactive HF mass spectrometer and Thermo Ultimate 3000LC. Chromatographic separation of the sample components was achieved a Zorbax Eclipse C18 column (1.8 μm × 0.25 mm × 100 mm). The mobile phases used were a mixture of water with 0.1% (V/V) formic acid (A) and acetonitrile (B). The gradient flow started with 5–30% B in 2 min, then 30% B at 7 min; followed by a linear increase to 78% B in 5 min, then 78% B at 14 min; followed by a linear increase to 95% B in 3 min, then 95% B at 17 min. The gradient returned to 5% B at 21 min, leading to a 25 min total run time. The flow rate was 0.3 ml min^−1^ with a typical injection volume of 2 μl. The column temperature was maintained at 30°C. Mass spectrometry was performed with ESI ionization: full scan MS‐DDMS2 acquisition mode; an ion transfer tube temperature of 350°C; spray voltage of 3.5 kV (+)/3.5 kV (−); sheath gas 45 arb; auxiliary gas 15 arb; a 100–1500 mass/charge m/z ratio range; resolution of 120,000 (full scan) and 60,000 (DDMS2).

### 2.4 GC–MS analysis

Sample extracts were analyzed with a Thermo Scientific TSQ‐8000 triple quadrupole mass spectrometer and a Trace GC 1300 gas chromatograph, which was equipped with a TriPlus AI 1310 autosampler (Thermo Fisher Scientific, San Jose, CA). Chromatographic separation was achieved with a J&W HP‐5MS capillary column (30 m × 0.25 mm, 0.25 μm film thickness). Helium (99.999% purity) was used as the carrier gas and maintained at a constant flow rate of 1.0 ml min^−1^. A sample volume of 1.0 μl was injected in the splitless mode with an ion source temperature of 300°C. The transfer line to tandem MS was maintained at 280°C. The column temperature was programmed from 70.0°C (held for 1.0 min) to 200°C at a rate of 15°C min^−1^, follow by 200°C to 260°C (held for 10 min) at a rate of 5 °C⋅min^−1^. The tandem MS was operated in the multiple reaction monitoring (MRM) mode with an electron energy of 70 eV and an emission current of 30 μA. Argon gas (Ar) was chosen as collision gas with a pressure of 1.5 mTorr. The full‐scan mode with the mixed standard solution at 1.0 μg⋅ml^−1^ was used to obtain the retention time (tR) and to select the most intense ions as optimal precursor ions.

### 2.5 DPPH scavenging capacity of the coffee samples

The DPPH scavenging capacity of the coffee samples was determined according to the literature method of DPPH assay (Deng et al., 2011; Hu et al., 2019): Rutin was used as a positive control. Sample mixtures were prepared by mixing 3.9 ml of DPPH (0.075 mM) and 0.1 ml of samples of different concentrations (0.1, 0.2, 0.3, 0.4, 0.5, 0.6, 0.7, 0.8, 0.9, and 0.1 mg/ml) and evaluated at 517 nm. The inhibition (*I*) was calculated using Equation (1)

(1) $$I\left(\%\right)=1-\left[\left(A_{o}-A_{s}\right)/A_{o}\right]\times 100\%$$

where *A*_s_ is the mixture of samples and DPPH, *A*_o_ is the DPPH.

### 2.6 ABTS assay of coffee samples

ABTS assay was performed by the modificatory method of Hu et al. (2019) and Magalhães et al. (2014). Test samples were prepared by mixing 2.0 ml of the ABTS^+^ radical solution (*A* = 0.70 ± 0.02, 734 nm) and 2.0 ml of 0.1 μg/ml sample and evaluated at 734 nm. Trolox was used as a standard (0, 2, 4, 6, 8, 10, 12, 14, 16, 18, and 20 [×10^−6^ mmol]) and the ABTS radical scavenging capacity was calculated by a calibration curve given by Equation (2)

(2) $$A=6.2085\times c-8.0807\left(R^{2}=0.9942\right)$$

### 2.7 FRAP assay of coffee samples

The FRAP assay was conducted using the reference method of Amamcharla and Metzger (2014). A mixture of 3.0 ml FRAP working reagent, prepared using TPTZ and FeCl_3_, was mixed with 100 μl sample solution and 300 μl deionized water. The sample mixture thus prepared was evaluated at 595 nm after being incubated at 37°C for 30 min. The calibration curve of Fe^2+^ standard solutions at different concentrations (0, 0.2, 0.4, 0.6, 0.8, 1.0, 1.2, 1.4, 1.6, 1.8, and 2.0 [×10^−4^ mol]) is given by Equation (3)

(3) $$A=0.6325\times c\left(R^{2}=0.9992\right)$$

### 2.8 Acetylcholinesterase inhibitory activity

Acetylcholinesterase (AChE) inhibitory activity of the samples was assayed by the spectrophotometric method developed by Ellman et al. (1961) with a slight modification. The reaction mixture (total 200 μl) containing phosphate buffer (pH 8.0), test extracts (50 μM), and acetylcholinesterase enzyme (0.02 U/ml) was incubated for 20 min (37°C). Then, the hydrolysis reaction was initiated by the addition of 40 μl solution containing 5,5′‐dithio‐bis‐(2‐nitrobenzoic) acid (DTNB, 0.625 mM) and acetylthiocholine iodide (0.625 mM) for acetylcholinesterase (AchE) inhibitory activity assay. The hydrolysis of acetylthiocholine was monitored at 405 nm every 30 s for 1 h. Tacrine was used as a positive control with a final concentration of 0.333 μM. All the reactions were performed in triplicate. The percentage inhibition (*I*) was calculated using Equation (4)

(4) $$I\left(\%\right)=\left(A_{o}-A_{s}\right)/A_{o}\times 100\%$$

where *A*_0_ is the activity of the enzyme without test extracts and *A*_s_ is the activity of enzyme with test extracts.

### 2.9 Statistical analysis

To establish the orthogonal partial least squares discriminant analysis (OPLS‐DA) model, a permutation analysis was carried out on the data with the number of tests set to 200; the differences between the two groups of data were analyzed as a whole to obtain the volcanic maps and variable importance in projection (VIP) prediction value distributions. The chemical compositions with VIP > 1, *p* < .01 and fold change (FC) > 4, or <0.25 were designated as significantly changed compounds. One‐way analysis of variance (ANOVA) with the least‐significant difference (LSD) method (*p* < .05) was applied to compare inhibition shown by different samples.

## 3 RESULTS

### 3.1 Chemical compositions of the coffee samples

**FIGURE 2:**
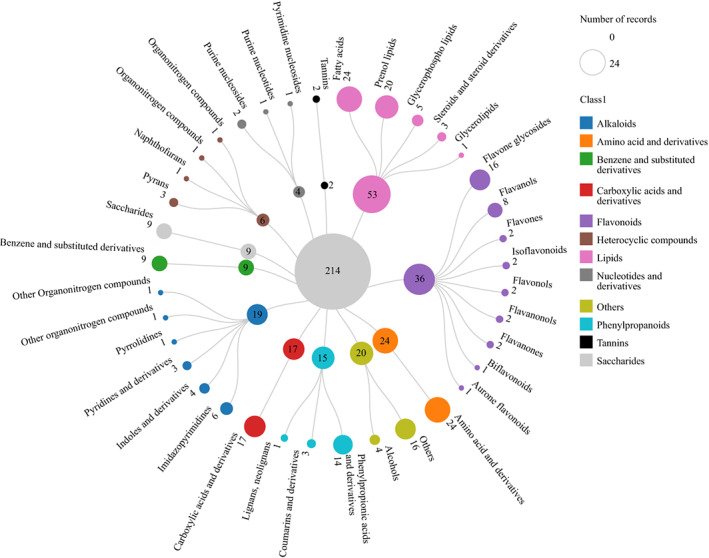
The number of chemical compositions in three different *C. Arabica* samples

The results of HPLC‐MS were processed and analyzed by Compound Discoverer Software 3.2 (Thermo Fischer scientific), Thermo mzCloud, and Thermo mzValut data. Based on the same extraction and analysis methods, the classes of chemical compositions of ACGB, ACF, and ACL were obtained and are shown in Figure 2. The results indicated that a total of 214 compounds have been confirmed in this study, and there were 12 classes of chemical compositions with differentiated distributions in three coffee samples including nucleotides and derivatives (0.012%–0.13%), tannins (0%–0.034%), flavonoids (0.021%–20.51%), alkaloids (6.20%–12.96%), benzene and derivatives (0.40%–1.20%), phenylpropanoids (37.98%–61.68%), amino acid and derivatives (2.54%–4.62%), lipids (0.35%–6.21%), heterocyclic compounds (0.091%–2.60%), carboxylic acids and their derivatives (2.36%–8.22%), saccharides (1.56%–12.77%), and others (6.24%–22.29%). Flavonoids and phenylpropanoids are their active components. Flavonoids comprised 36 compounds such as rutin, luteolin, catechin, (−)‐epicatechin, procyanidin A2, procyanidin B1, procyanidin B2, procyanidin C1, quercetin, kaempferol, fisetin, and others. Phenylpropanoids comprised 19 compounds such as chlorogenic acid, caffeic acid, ferulic acid, 3‐*O*‐feruloyl‐quinic acid, 3,4‐di‐*O*‐caffeoylquinic acid, and others.

**FIGURE 3:**
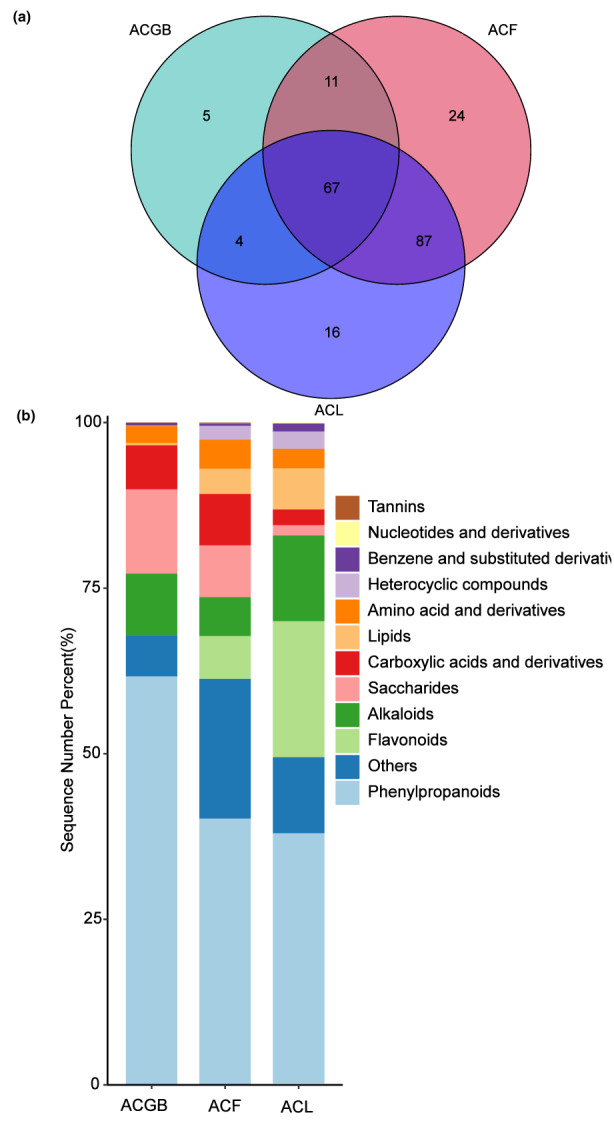
Venn map of the number of chemical compositions in three different *C. Arabica* samples (a), percentage of chemical compositions in three different *C. Arabica* samples (b)

Figure 3a is a Venn map showing numbers of chemical compositions that were either shared or unique. The Venn map showed that 5, 24, and 16 chemical compositions were unique to ACGB, ACF, and ACL, respectively. Besides, compared to chemical compositions in ACGB (87 compounds), the chemical compositions of ACF and ACL were richer, identified with 189 and 174 chemical compounds, respectively. In addition, the three coffee samples analyzed exhibited similar chemical compositions, 67 compounds were common in all the three coffee samples as shown in Figure 3a. In addition, common chemical compositions of ACF and ACL were found to be 154. However, the common chemical compositions of ACF and ACGB, ACL and ACGB only had 78, 71, respectively.

Moreover, Figure 3b shows the proportions of each class of chemical compositions in each *C. Arabica* sample in different colors, and the lengths of the bars in the bar chart indicate the various levels of chemical compositions. Phenylpropanoids were identified to be the highest percentages out of 12 classes of chemical components in three samples, which ranged from 37.977% to 61.683%. Moreover, the percentages of phenylpropanoids in ACF and ACL were lower than in ACGB. Flavonoids were identified as primary components in ACL and ACF, and the percentages were 20.51% and 6.82%, respectively; while it was only 0.021% in ACGB. Meanwhile, the percentage of alkaloids in ACL was 12.96%, which was higher than ACGB (9.24%). However, the value of alkaloids in ACF was 6.20% which was lower than ACGB. Compared with ACF and ACL, the percentages of saccharides, and carboxylic acids and their derivatives were significantly different, which were 12.77% and 6.63%, respectively, in ACGB. Then, saccharides, and carboxylic acids and their derivatives were found to be the important and main compositions in ACF with 8.26% and 8.22%, respectively, and were lowest in ACL with 1.56% and 2.36%, respectively. Nucleotides and their derivatives, tannins, benzene and derivatives, amino acid and derivatives, lipids, and heterocyclic compounds were low in content in the three samples.

**FIGURE 4:**
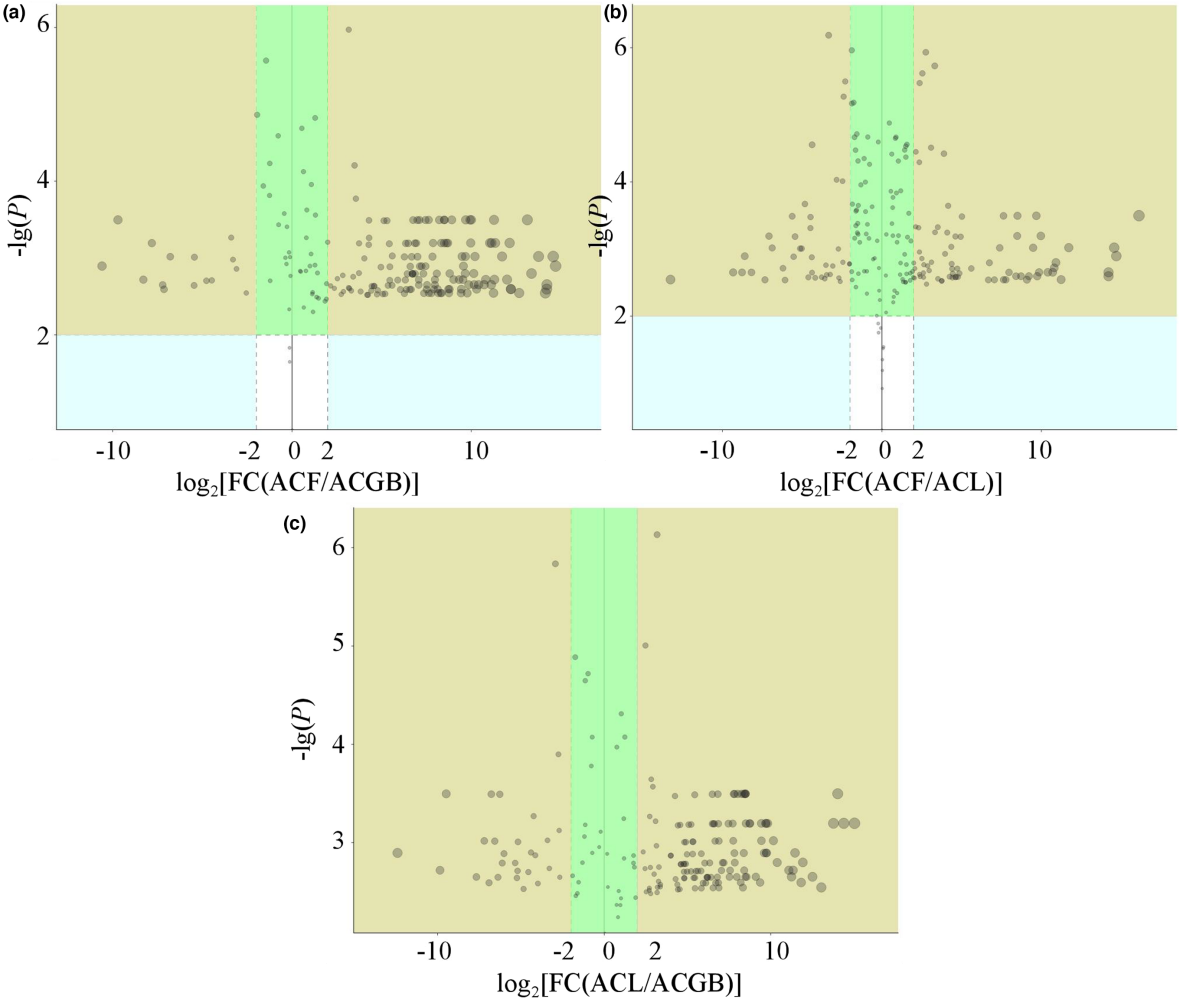
Volcano plot of the contribution of chemical compositions in ACF and ACGB (a), volcano plot of the contribution of chemical compositions in ACF and ACL (b), volcano plot of the contribution of chemical compositions in ACL and ACGB (c)

**FIGURE 5:**
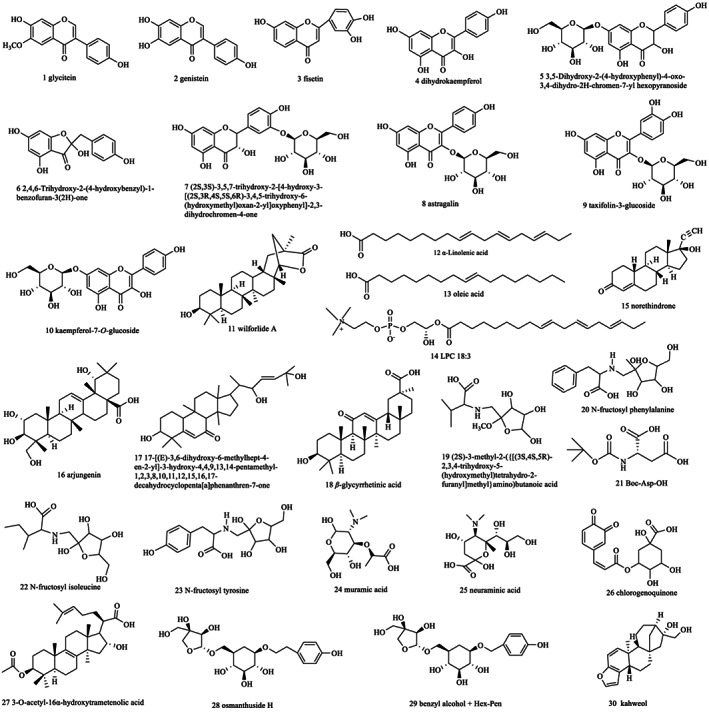
The structures of 30 differentially changed chemical compositions in three different coffee samples

Under the conditions of VIP > 1.0, *p* < .01, FC > 4 or FC < 0.25, Figures 4a–c were selected to compare the differentially changed chemical compositions (Li et al., 2022; Wang et al., 2021) in ACF and ACGB, ACF and ACL, ACL and ACGB, respectively. Meanwhile, 30 chemical components, including 10 flavonoids (1–10), 8 lipids (11–18), 5 amino acid and derivatives (19–23), 4 carboxylic acids and their derivatives (24–27), 2 saccharides (28 and 29), and 1 heterocyclic compound (30) were identified as differentially changed chemical compositions (VIP > 1.0, *p* < .01, FC > 4 or FC < 0.25) in these three coffee samples, and structures of these chemical compounds are shown in Figure 5.

**FIGURE 6:**
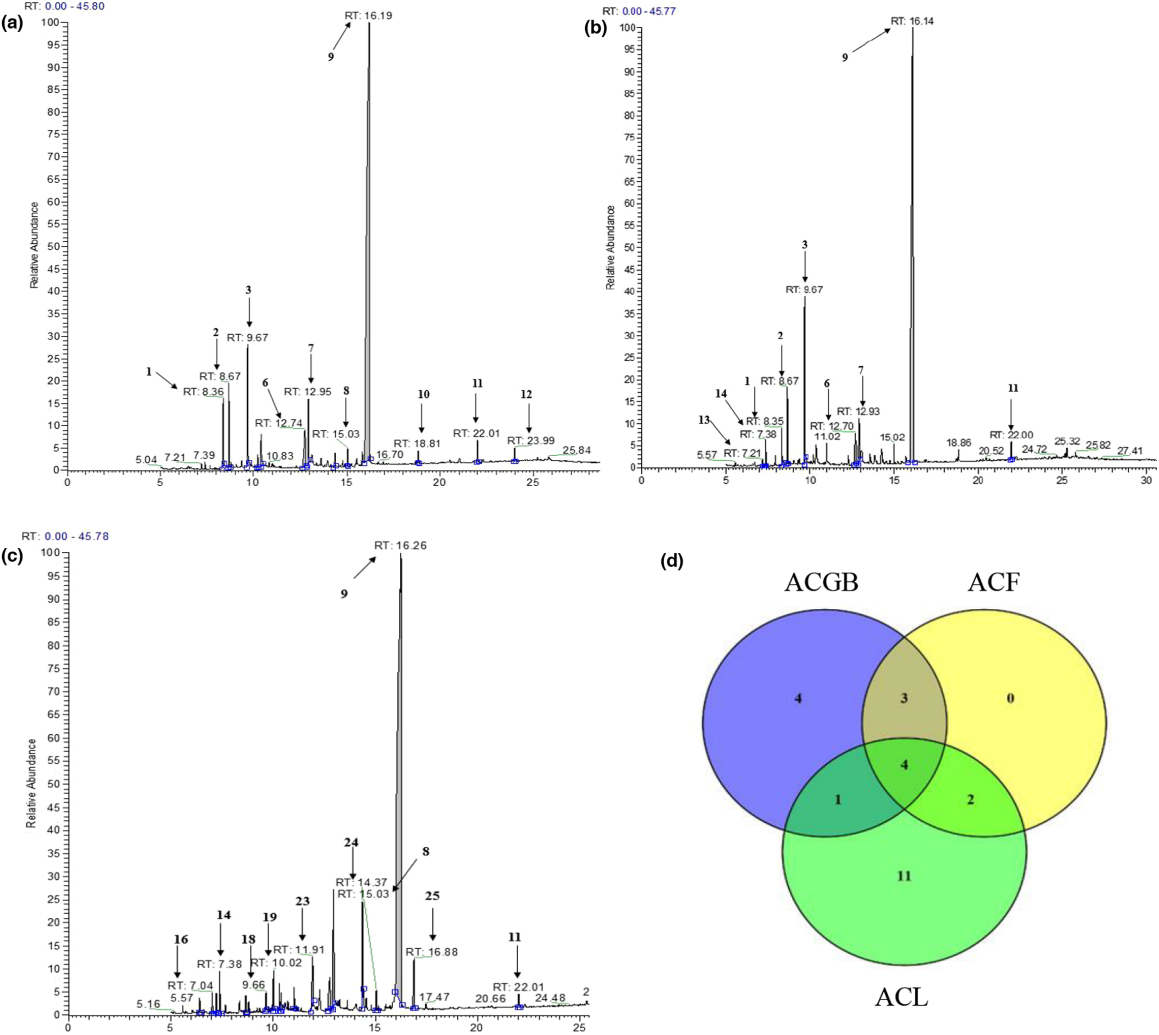
The diagrams of aroma compositions by GC–MS in ACGB (a), ACF (b), ACL (c), venny map of the number of aroma compositions in three different *C. Arabica* samples (d)

The Thermo Scientific automatic selected reaction monitoring (AutoSRM) software was used to process product‐ion scan with a range of different collision energies. A total of 25 volatile compounds were identified by GC–MS as shown in Table 1 and were marked in Figures 6a–c. The spectrograms of 7 volatile compounds (4, 5, 15, 17, 20, 21, and 22) were unclear for their low contents. Peak 9 was found to be the primary component in these three coffee samples with high intensities and was confirmed to be as caffeine accounting for the maximum area in all compounds. Compared to ACGB, ACL was richer in volatile compounds as 18 volatile compounds were detected in ACL; then there were 12 in ACGB, and 9 in ACF, respectively. These compounds are marked in Figure 6d. Four compounds were common in all the three coffee samples namely caffeine (78.21%–80.49%, %Area), n‐hexadecanoic acid (3.02%–5.66%, %Area), quinic acid (2.25%–3.70%, %Area), and 2,2′‐methylenebis[6‐(1,1‐dimethylethyl)‐4‐methyl‐phenol] (0.49%–1.13%, %Area).

**TABLE 1:** Volatile compositions of coffee samples by GC–MS

Number	Compounds	%area		
		ACGB	ACF	ACL
1	2‐methoxy‐4‐vinylphenol	2.13	1.93	–
2	1,2‐diphenyl‐1‐methyl‐2‐trimethylsilyl‐ethylene	2.03	2.65	–
3	2‐hydroxy‐6‐methyl‐benzaldehyde	3.56	7.23	–
4	6‐methyl‐5‐phenyl‐hept‐5‐en‐2‐one	0.32	–	–
5	bis[(2Z)‐hex‐2‐en‐1‐yloxy](dimethyl)silane	1.24	–	–
6	Quinic acid	3.70	2.57	2.25
7	n‐hexadecanoic acid	3.07	3.02	5.66
8	Dibutyl phthalate	0.57	–	0.53
9	Caffeine	80.48	80.19	78.21
10	bis(2‐ethylhexyl) ester‐hexanedioic acid	0.50	–	–
11	2,2′‐methylenebis[6‐(1,1‐dimethylethyl)‐4‐methyl‐phenol	0.99	1.13	0.49
12	1,4‐benzenedicarboxylic acid, bis(2‐ethylhexyl) ester	0.58	–	–
13	Catechol	–	0.29	0.61
14	2,3‐dihydro‐benzofuran	–	0.99	0.89
15	Phenylethyl alcohol	–	–	0.49
16	Methyl salicylate	–	–	0.63
17	3,6‐dimethoxy‐9,10‐dimethyl‐phenanthrene	–	–	0.33
18	2‐hydroxy‐5‐methylbenzaldehyde	–	–	0.45
19	4′‐hydroxy‐acetophenone	–	–	0.79
20	3,5,3′,5′‐Tetramethyl‐N4‐propyl‐biphenyl‐4,4′‐diamine	–	–	0.54
21	5,5‐Dimethyl‐1‐oxa‐5‐cyclopentanone‐9	–	–	0.44
22	D‐allose	–	–	0.89
23	4‐propyl‐1,3‐benzenediol	–	–	2.11
24	Phytol	–	–	2.70
25	Theobromine	–	–	1.99

### 3.2 Antioxidant activity

The three models of testing the antioxidant activities of three coffee samples were based on different principles and the results are shown in Table 2. The DPPH assay, as a popular model estimating the antioxidant capacity of samples, is highly efficient and sensitive. Among these three samples, ACL showed the highest DPPH radical scavenging capacity with IC_50_ 0.491 ± 0.0148 mg/ml, followed by ACGB (IC_50_, 0.674 ± 0.088 mg/ml), and ACF (IC_50_, 0.815 ± 0.098 mg/ml). The ABTS assay is another common method for estimating the antioxidant capacity of samples. In the ABTS assay, ACF showed the highest ABTS radical scavenging activity with 36.413 ± 1.523 mmol trolox/g Ex. In addition, the other two samples had very low activity. ABTS radical scavenging activity value for ACGB was 3.115 ± 0.105 mmol Trolox/g Ex. ACL showed the lowest antioxidant efficacy with 1.891 ± 0.244 mmol Trolox/g Ex. The principle of FRAP assay is to reduce Fe^3+^‐TPTZ to Fe^2+^‐TPTZ. However, the highest antioxidant efficacy was shown by ACGB (0.398 ± 0.020 mmol Fe^2+^/g Ex). The FRAP value for ACL was 0.237 ± 0.014 mmol Fe^2+^/g Ex. ACF showed the lowest antioxidant efficacy with 0.202 ± 0.044 mmol Fe^2+^/g Ex.

**TABLE 2:** Antioxidant activities of methanolic extracts of *C. Arabica* flowers (ACF), leaves (ACL) and green coffee beans (ACGB)

Samples	ACF	ACL	ACGB
DPPH (IC_50_, mg/ml)	0.815^b^ ± 0.098	0.491 ^e^ ± 0.015	0.674^d^ ± 0.088
ABTS (mmol Trolox/g)	36.413^a^ ± 1.523	1.891^c^ ± 0.244	3.115^b^ ± 0.105
FRAP (mmol Fe^2+^/g)	0.202^c^ ± 0.044	0.237^b^ ± 0.014	0.398^a^ ± 0.020

*Note*: Different lowercase letters in the same column indicate significant differences (*p* < .05).

With rutin as a positive control, the value of IC_50_ in DPPH radical scavenging assay was 0.129 ± 0.0046 mg/ml, ABTS radical scavenging activity value was 23.671 ± 1.868 mmol Trolox/g Ex, and FRAP value was 0.302 ± 0.022 mmol Fe^2+^/g Ex. Compared with rutin, the antioxidant activities of these samples were relatively lower. However, ACL exhibited the strongest DPPH radical scavenging capacity and ACF showed the strongest ABTS radical scavenging activity. Therefore, ACL and ACF had potential antioxidant ability against oxidative stress.

### 3.3 Acetylcholinesterase inhibitory activity

Compared with Tacrine, ACL showed weak acetylcholinesterase inhibitory activity with an inhibition ratio of 25.18 ± 2.96%. While ACGB and ACF did not show acetylcholinesterase inhibitory activity, values of inhibition ratio being 0.95 ± 6.48% and 0.83 ± 4.04%, respectively.

## 4 DISCUSSION

The chemical constituents of coffee, which are the basis of different biological activities of coffee and contribute to the characteristic flavor, are large in number, including alkaloids, phenolic acids, flavonoids, etc. (Shen et al., 2020). Substantiating the information available in literature, caffeine and chlorogenic acid were confirmed in this study as the main classic compounds in coffee. The contents of caffeine, chlorogenic acid, and quinic acid were analyzed semi quantitatively using 2‐amino‐3‐(2‐chloro‐phenyl)‐propionic acid as an interior label compound. Caffeine was relatively high in content compared to other compounds, the values are as follows: 139.198 μg/ml (ACGB), 128.961 μg/ml (ACF), and 246.934 μg/ml (ACL). The content of chlorogenic acid was the highest in ACGB with a value of 1028.431 μg/ml and then in ACF it was 1505.595 μg/ml. While quinic acid content in ACL was the highest with a value of 694.289 μg/ml, followed by chlorogenic acid with 407.506 μg/ml Neochlorogenic acid (461.472 μg/ml), and α,α‐trehalose (403.592 μg/ml) were high content compounds in ACGB, after chlorogenic acid. Quinic acid (649.289 μg/ml) and 4‐(2,4‐dichlorophenyl)‐7‐(3.4‐dimethoxyphenyl)‐2‐methyl‐5‐oxo‐1,4,5,6,7,8‐hexahydroo‐3‐quinolinecarboxylate ethyl (619.708 μg/ml) were high content compounds in ACF, after chlorogenic acid. Mangiferin (338.210 μg/ml) was high content compound in ACF, after quinic acid, and chlorogenic acid.

Phenolic acids and their derivatives from coffee mainly include mono‐, di‐caffeoylquinic acid, feruloylquinic acid, *p*‐coumaroylquinic acid, and their methyl esters (Shen et al., 2020). This study found that phenylpropanoids and their derivatives including quinic acid, chlorogenic acid, caffeic acid, ferulic acid, 4,5‐dicaffeoylquinic acid, 3,4‐di‐*O*‐caffeoylquinic acid, 3‐*O*‐feruloylquinic acid are the highest quantity components in chemical composition of coffee. Flavonoids including catechins, anthocyanin, myricetin, fisetin, patuletin, luteolin, apigenin, and quercetinare are widely present in coffee leaves (Chen, 2019; Júnior et al., 2012; Ngamsuk & Huang, 2019; Patay et al., 2016). Flavonoids (20.513% in ACL and 6.818% in ACF) including flavones, isoflavonoids, flavanols, flavanonols, and flavone glycosides, such as rutin, kaempferol, quercetin, catechin, epicatechin, procyanidin A_2_, procyanidin B_1_, procyanidin B_2_, and procyanidin C_1_ are also present. Moreover, these compounds have been confirmed to show an antioxidant effect. Conversely, 0.0262% flavonoids were detected in ACGB, including epigallocatechin and procyanidin A_2_. Thirty distinct characteristic compounds obtained from these three coffee samples would help in the rapid analysis of the coffee samples. Terpenes as a type of characteristic constituents in *C. Arabica* include the skeletons of ent‐kaurane, kahweol, villanovane diterpenoid, ent‐kaurane diterpenoid glucosides, dammarane, and pentacyclic triterpene (Shen et al., 2020). However, kahweol belonging to heterocyclic compounds was confirmed as the only terpene in this study. This may be attributed to the fact that new compounds with unknown structures are usually recovered in low concentrations; hence, they are not included in Thermo mzCloud and Thermo mzValut data.

The antioxidant properties of food products are considered as parameters of nutritional quality (Carlsen et al., 2010; Yang et al., 2011). Coffee contains multiple active components, including caffeine, phenolic acids, and flavonoids. The introduction of coffee by‐products as a novel food in the food sector still needs many efforts. Pinheiro et al. (2021) verified that the extracts of coffee flowers have antioxidant potential activities by ABTS assay. Fu et al. (2021) evaluated the antioxidant potential of the extracts from *C. Arabica* husk by quenching free radical scavenging assay, reducing power, and ORAV assay. Ngamsuk and Huang (2019) found that the methanolic extract from different leaves of *C. Arabica* showed high antioxidant activities, fresh young (92.93 ± 0.51%), fresh mature (92.24 ± 0.95%), dried young (95.01 ± 0.44%), dried mature (93.40 ± 0.70%), respectively. ACL showed DPPH radical scavenging capacity with EC_50_ of 0.491 ± 0.148 mg ml^−1^ and acetylcholinesterase inhibitory activity with an inhibition ratio of 25.18 ± 2.96%. Meanwhile, ACF showed ABTS radical scavenging activity with 36.413 ± 1.523 mmol Trolox/g Ex.

In addition, coffee leaves have been used to prepare tea‐like drinks through leaf steaming, rolling, and drying production methods for a long time (Ratanamarno & Surbkar, 2017). Alternatively, the coffee leaves are also fermented and roasted as well as used as medicine in some originating countries. Because they contain multiple bioactive compounds including terpenes, tannins, phenolic acids, flavonoids, phytosterols, and carotenoids, which are related to their diverse potential bioactive effects. Moreover, coffee leaves were determined as a traditional food by the EFSA (European Food Safety Authority) in the context of a novel food notification from a third country. These results provide sufficient support to the use of ACL and ACF as novel potential food materials.

Moreover, many chemical compounds, such as flavonoids, polysaccharides, triterpenoids, also have acetylcholinesterase inhibitory activity (Li et al., 2020; Liu et al., 2019; Xu et al., 2022). In the same extraction conditions, ACL was richer in chemical compounds compared to ACGB and ACF. Therefore, ACL showed higher activity than ACGB and ACF. However, the potentially active compounds were not pure and hence further studies are needed.

## 5 CONCLUSIONS

Chemical compounds obtained from plant‐based beverages are not only related to the special flavor of food but also contain various nutritional and functional values. The antioxidant property of food products is considered to be an important parameter of nutritional quality. Moreover, neuroprotective activity is a special bioactivity of coffee, especially for the treatment of AD. Therefore, the comparison of antioxidant activity and acetylcholinesterase inhibitory activity of coffee by‐products with ACGB was important for evaluating their use as potential novel foods. A total of 214 chemical compositions belonging to 12 classes were detected by HPLC‐MS, with a majority of them being active in multiple areas. Phenylpropanoids account for the highest percentage of coffee's chemical composition. However, when compared with ACGB, flavonoids, alkaloids, saccharides, and carboxylic acid and their derivatives were significantly different in content. Thirty distinct characteristic compounds were also identified in three coffee samples. Eighteen characteristic compounds were identified by GC–MS. Antioxidant effects of ACL and ACF were higher in value than ACGB. ACL showed weak acetylcholinesterase inhibitory activity. Therefore, ACF and ACL are potential and promising sources of bioactive compounds, with a scope of further studies on their human health effects. Taken together, results obtained in this study can be used as a reference to understand the chemical compositions of coffee by‐products, and provide relevant data support for the development and utilization of coffee by‐products as novel food items.

## CONFLICT OF INTEREST

All authors declare that they have no known competing financial interests or personal relationships that could have appeared to influence the work reported in this paper.

## Data Availability

Data available on request from the authors: The data that support the findings of this study are available from the corresponding author upon reasonable request.
